# Differences in the distribution of attention to trained procedure between finders and non-finders of the alternative better procedure

**DOI:** 10.3389/fpsyg.2022.934029

**Published:** 2022-08-23

**Authors:** Yuki Ninomiya, Hitoshi Terai, Kazuhisa Miwa

**Affiliations:** ^1^Global Research Institute for Mobility in Society Institutes of Innovation for Future Society, Nagiya University, Nagoya, Japan; ^2^Faculty of Humanity-Oriented Science and Engineering, Kindai University, Fukuoka, Japan; ^3^Graduate school of Informatics, Nagiya University, Nagoya, Japan

**Keywords:** Einstellung effect, mental set, problem solving, curiosity (curiositas), eye movement analysis

## Abstract

The human ability to flexibly discover alternatives without fixating on a known solution supports a variety of human creative activities. Previous research has shown that people who discover an alternative procedure relax their attentional bias to information regarding the known solutions just prior to the discovery. This study examined whether the difference in the distribution of attention between the finders and non-finders of the alternative procedure is observed from the phase of solving the problem using the trained procedure. We evaluated the characteristics of the finders' distribution of attention in situations where problem solving using a trained procedure was successful. This aspect has been little examined in previous research. Our study obtained empirical evidence for the fact that, compared to non-finders, finders pay more attention to information unrelated to the trained procedure acquired through knowledge and experience, even time when using a trained procedure. We also confirmed that this difference does not exist from the beginning of the task, but emerges during repeated use of familiar procedures. These findings indicate that in order to find an alternative procedure, one should not only divert attention from a familiar procedure just before the discovery but also pay a certain amount of attention to information unrelated to the familiar procedure even when the familiar procedure is functioning well.

## Introduction

Cognitive flexibility is the human ability to adapt to problems that require the use of new methods or knowledge, or changes in existing coping strategies (Cañas et al., 2003; Braem and Egner, 2018; Ohlsson, 2018). This ability supports a variety of human creative activities such as creative and scientific discovery (Jansson and Smith, 1991; Neroni et al., 2017), experts' skills (Bilalić et al., 2008a,b, 2010), and entrepreneurs' innovativeness (Sahai and Frese, 2019).

The Gauss (1777–1855) calculation anecdote (Hayes, 2006) is a good example. In this anecdote, Gauss as a child was asked by his teacher to add up the numbers from 1 to 100. Gauss found the solution more easily by adding 1 and 100 and then multiplying by 100/2. The sum from 1 to 100 is obtained by performing 1 + 100, 2 + 99…50 + 51 in a sequence. Since every sum of each step always equals 101, the total sum can be obtained by performing 101 × 50. In other words, Gauss solved the problem efficiently with one simple addition (1 + 100) and one multiplication (101 × 100/2) in very few steps, whereas the procedure of adding sequentially from 1 to 100 requires 99 steps. In order to discover a more efficient method, as Gauss did, we need to dismiss the familiar solution (adding in order from 1) and try to discover a better alternative solution. There are many theories about the truth of this anecdote (Hayes, 2006). However, this example suggests that flexibility, the ability to dismiss familiar solutions and explore more efficient ones, is important in creative discovery. In this study, we examined the process of discovering better solutions by comparing participants who discovered them and those who did not.

Studies suggest that it is difficult to reject familiar procedures and discover better solutions, even when familiar procedures are complex and effortful (Luchins, 1942; Luchins and Luchins, 1950; Schultz and Searleman, 2002; Bilalić et al., 2008a,b, 2010; Haager et al., 2014; Huang et al., 2019). Such a phenomenon is called the Einstellung (set) effect. The Einstellung effect is an occurrence in which a solution is recalled based on prior experiences or knowledge that hinder the discovery of other, more sophisticated resolutions.

The water jar problem (Luchins, 1942; Luchins and Luchins, 1950) is a typical, traditional task that demonstrates this effect. The goal of this task is to draw the target amount of water using three different-sized water jars, A, B, and C. This task consists of three types of trials: set, critical, and inspection. In the set trial, a problem that can be solved only by a specific procedure (e.g., B–A−2 C) is repeatedly presented, and participants learn this procedure (this procedure is called a trained procedure). Next, a critical trial can be solved by two procedures: a trained procedure and a simpler procedure such as C–A (the procedure that is more efficient and easier than the trained procedure is called an alternative procedure). There is no feedback that there is something wrong with the trained procedure because the goal amount of water can be obtained using the trained procedure. Therefore, it is demonstrated that finding an alternative procedure is difficult because the trained procedure is applied (Luchins, 1942; Luchins and Luchins, 1950). The last trial, the inspection trial, is a trial that can be solved only by the alternative procedure. The participant is forced to explore the new procedure as the problem cannot be solved using the trained procedure. Nevertheless, even in this trial, it is demonstrated that the discovery rate is lower and the time required for discovering the alternative procedure is longer than when the participants have not learned the trained procedure (Luchins, 1942; Luchins and Luchins, 1950).

The Einstellung effect has been repeatedly demonstrated in various domains and contexts (variations in cognitive tasks: Ellis and Reingold, 2014; Huang et al., 2019; functional fixation: Duncker, 1945; design fixation: Jansson and Smith, 1991; Neroni et al., 2017; expertise: Ricks et al., 2007; Bilalić et al., 2008a,b, 2010; Sheridan and Reingold, 2013; magic tricks: Thomas et al., 2015, 2018; mathematics: Chesney et al., 2013; etc.). The tendency to continue to use a mental or behavioral set, such as a trained procedure, can also be positioned as a personality factor called rigidity (Schultz and Searleman, 2002).

The mechanism behind the Einstellung effect is not yet completely clear, but explanations have been associated with confirmation bias and the spreading activation of memory (Bilalić et al., 2008b; Thomas et al., 2018; Blech et al., 2020; Navarre et al., 2022). When we test a hypothesis, we tend to look for evidence that confirms it rather than for evidence that rejects it (Wason, 1960, 1968; Griggs and Cox, 1982). This bias is referred to as confirmation bias. This tendency causes people to ignore evidence that contradicts their hypothesis. Bilalić et al. (2008b) explained that the Einstellung effect occurs because people apply trained procedures that come to mind immediately due to this tendency. In addition, in the explanation based on the spreading activation of memory, it is explained that recalling a trained procedure activates the semantic field associated with the procedure.

Furthermore, this activation of the related knowledge is explained to be the cause of the fixation to the trained procedure even after it is rejected (Thomas et al., 2015, 2018; Blech et al., 2020). This explanation illustrates one aspect of the Einstellung effect, which is the tendency to fixate on a trained procedure even when people are aware that it is not valid.

In discussing the discovery of alternative procedures under the Einstellung effect, it is necessary to distinguish between finding an alternative procedure in situations where the problem “can” be solved by a trained procedure such as in critical trials, and finding an alternative procedure in situations where the problem “cannot” be solved by a trained procedure such as in inspection trials. This is because problem-solving process leading to the discovery is considered to be different between the former and the latter.

The major difference between the former and the latter problem-solving process is whether or not the need to change the trained procedure is feedback. In the former, since the problem can be solved by a trained procedure, the necessity to change the procedure is not explicitly stated. In the latter case, since problem solving using the trained procedure fails, the participants are forced to abandon the trained procedure. Therefore, the discovery of an alternative procedure is inevitably more likely to occur in the latter than in the former situation (Chesney et al., 2013; Sheridan and Reingold, 2013).

The latter process is discussed based on the relationship between the failure of the trained procedure and the change in the search space (Thevenot and Oakhill, 2005, 2008; Chesney et al., 2013). For example, Chesney et al. (2013) explained the finding of an alternative procedure in relation to the representational change theory of insight (Knoblich et al., 2001). They explained that in situations where the Einstellung effect is observed, a trained procedure is recalled as the initial representation, and therefore, in order to discover an alternative procedure, the initial representation must be changed. They also pointed out the importance of feedback of failure in changing initial representations, in that the Einstellung effect is particularly harmful when there is no feedback that the trained procedure is wrong. This point is also supported by studies using eye movement as a measurement, which show that when a task cannot be solved by a familiar trained procedure, attentional bias toward areas related to this procedure is alleviated, regardless of whether the task is performed by an expert or non-expert (Sheridan and Reingold, 2013). In other words, the process of finding an alternative procedure in a situation where a problem cannot be solved by a trained procedure can be described as a process that leads to discovery by diverting attention from the relevant area of the trained procedure by the feedback of failure.

In the former case, where the problem can be solved by the trained procedure, this explanation cannot be applied. This is because, in this situation, the problem can be solved by the trained procedure, and there is no feedback on the need to change this procedure. Therefore, in order to find an alternative procedure, it is necessary to spontaneously abandon the trained procedure and search for another procedure.

The elucidation of such problem-solving processes that lead to alternative solutions in situations where there is no feedback of failure is important for understanding the high degree of flexibility of humans. For example, it has been shown that highly skilled experts, called spar experts, might find alternative better solutions even when the problem can be solved by a trained procedure (Bilalić et al., 2008a,b, 2010; Sheridan and Reingold, 2013). In other words, in order to elucidate a high degree of flexibility similar to that of experts, it is necessary to explain the process of finding the alternative procedure when problem solving is successful using a trained procedure. The importance of examining the process of finding in this situation is highlighted, not only in studies of cognitive flexibility but also in studies related to curiosity (Gottlieb et al., 2013; Hagtvedt et al., 2019). For example, Hagtvedt et al. (2019) showed that spontaneous exploration based on curiosity promotes the generation of creative ideas and products. Nevertheless, no research has categorized the success or failure of such a trained procedure, and in particular, there is little clarification of the mechanism of finding an alternative procedure in a situation where the problem can be solved by a trained procedure.

In this study, we called the participants who found the alternative procedure in a situation where the problem was solved by the trained procedure, finders. We also examined the nature of the problem-solving process of the finders in contrast to the non-finders who fixated on the trained procedure and could not shift to the alternative procedure.

Research with experts can provide hints about the nature of the problem-solving process of finders. Bilalić et al. (2008a,b) conducted a chess-based Einstellung task on chess experts. The goal of the task was to checkmate in as few moves as possible. An important feature of this task was that it included two procedures: a familiar procedure that required five moves to checkmate, and an optimal, but unfamiliar procedure that required three moves to checkmate. As the familiar procedure was immediately recalled by chess experts, this procedure corresponded to the trained procedure, while the optimal procedure corresponded to the alternative procedure.

They illustrated using this task that even experts have difficulty finding an alternative procedure in situations where the solution is achieved by a trained procedure (Bilalić et al., 2008a,b). In addition, analysis of eye movements during the task showed that the attention of participants who failed to find the alternative procedure was biased toward regions related to the trained procedure, although they reported that they were searching for other solutions (Bilalić et al., 2008b). In contrast, experts who were able to discover the alternative procedure reduced the attentional bias toward the region associated with the trained procedure (Bilalić et al., 2008b; Sheridan and Reingold, 2013). Sheridan and Reingold (2013) showed that experts who found the alternative procedure allocated less attention to areas related to the trained procedure toward the end of the task, compared to those who did not find the alternative procedure. In summary, the findings suggest that finders gradually shift their attention from areas related to the trained procedure, to finding the alternative procedure.

This study aimed to extend the findings of previous studies by clarifying the timing in which the difference in the distribution of attention between finders and non-finders was observed. To summarize the previous studies, once a trained procedure is learned and a mental set is formed, this procedure is immediately recalled and applied (Bilalić et al., 2008a; Sheridan and Reingold, 2013). This is common to both finders and non-finders. At a certain point, finders can distract their attention from the area related to the trained procedure and direct their attention to other areas (Bilalić et al., 2008a,b; Sheridan and Reingold, 2013). In order to explain this difference between finders and non-finders, it is important to examine whether the difference in the distribution of attention between them can be seen from the stage of applying the trained procedure. If a difference is observed at this stage, it means that there is not only a difference in the ability of the finders and the non-finders to direct their attention to other procedures (distract their attention from the trained procedure) but also a difference in the intensity of their fixation and the information to which attention is directed from the stage of using the trained procedure before that.

Previous studies used tasks that were not segmented by the used procedure and therefore could not clearly identify from what point participants stopped using a trained procedure (Bilalić et al., 2008b; Sheridan and Reingold, 2013). Hence, even when differences in the distribution of attention were observed, it was not clear whether they appeared during the phase of solving the problem with the trained procedure or during the phase involving processes after discovery, such as noticing the alternative procedure and confirming its effectiveness. For example, Sheridan and Reingold (2013) split a trial in which an alternative procedure was discovered in four sections and showed that the difference in the distribution of attention between finders and non-finders increased later. However, this method cannot exclude the possibility that an alternative procedure had already been discovered at the time the difference was observed. In other words, it cannot be known whether the finders were solving the problem with a trained procedure at the time the difference was observed.

This study solves this issue by considering trials with the water jar problem (Luchins, 1942; Luchins and Luchins, 1950) as a single segment. Trials with the water jar task are distinguished into trials that first reported an alternative procedure (called the finding reporting trial) and earlier trials that reported a trained procedure. This ensured that participants in the experimental procedure were using the trained procedure on trials prior to the finding reporting trial. This ensured, at least in terms of the experimental procedure, that participants were using the trained procedure on trials prior to the finding reporting trial. It is not surprising that the distribution of the finder's attention differed from that of the non-finder in the finding reporting trial and subsequent trials. This is because participants solved the problem using different procedures (the finders used an alternative procedure, and the non-finders use a trained procedure). By contrast, in the trials prior to the finding reporting trial, the finder and non-finder used the same procedure to solve the problem. Therefore, we could examine whether there were differences in the degree of fixation and the information to which attention was directed from the phase in which the trained procedure was used, by confirming the difference in the distribution of attention in the trials before the finding reporting trial. Confirming this difference was the primary interest of this study.

Furthermore, the present study examined how this difference in the distribution of attention was caused. In previous studies using the subject of chess, only experts could find an alternative procedure in situations where a trained procedure could solve the problem (Bilalić et al., 2008a,b, 2010; Sheridan and Reingold, 2013). Experts have a wealth of domain-specific knowledge and skills compared to novices, allowing them to adopt diverse and sophisticated approaches to problems in their area of expertise (Ericsson and Lehmann, 1996; Ericsson, 2006). Therefore, it is possible that there was a difference in the tendency to distribute attention between experts and novices such as the number and quality of strategies and sophisticated skills, even before the task began.

Accordingly, this study examined whether differences can be observed in the participants' inherent tendency to distribute attention. If there is a difference in the distribution of attention as a trait of the participants, this difference should be observed from the early stage of the task, not just before finding the alternative procedure. In examining this question, the comparison between experts and novices is not an appropriate subject. This is because experts undergo significant long-term training compared to novices (Ericsson and Lehmann, 1996), and factors such as differences in the amount of training are confounded. In other words, in order to compare the distribution of attention as a trait of the participants, it was necessary to compare participants with the same degree of training. In the present study, we checked whether the difference in the distribution of attention in trials prior to the finding reporting trial also occurred in the early stages of the task, i.e., in the early stages of the set trial. This allowed for the comparison of participants with the same amount of training on a particular problem. Confirmation of this difference was the second interest of this study.

In summary, this study examined whether there was a difference in the distribution of attention during the phase of problem solving using the trained procedure, i.e., the trial before the finding reporting trial. If there was a difference in the distribution of attention in the trial prior to the reporting finding, we confirmed whether the difference was observed from the initial stage of the task, i.e., the first trial of the set trial. Previous studies have revealed the features of the distribution of attention in the process of finding an alternative procedure by using eye movement measures to observe visual searches for areas involved in trained/alternative procedures on the display (Bilalić et al., 2008a,b, 2010; Sheridan and Reingold, 2013). Other problem-solving studies, such as those examining insight, have also demonstrated the effectiveness of eye movement measurements in examining processes during problem solving (Knoblich et al., 2001; Grant and Spivey, 2003; Ellis et al., 2011; Bilalić et al., 2021). Therefore, we also examined the distribution of attention to regions involved in trained/alternative procedures during the task by capturing participants' eye movements.

## Methods

### Participants

Sixty undergraduates from Nagoya University participated in the experiment lasting for 90 min; they were paid ¥2,000 as remuneration. The experiments were conducted individually. Informed consent was obtained from the participants before starting the experiment. This experiment was approved by the ethics board of the institution to which we belong.

### Equations

**Figure 1:**
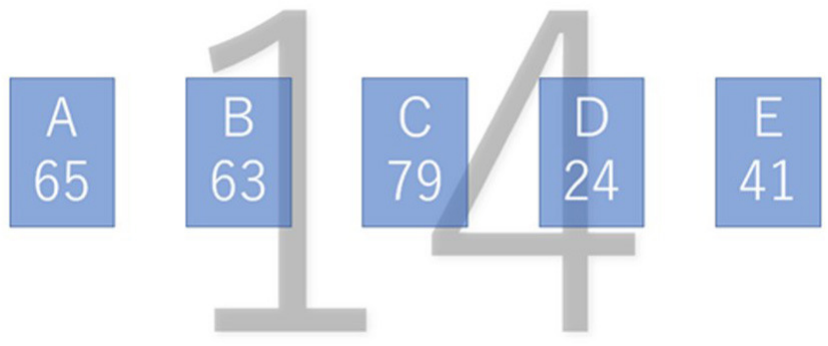
A sample of the stimulus.

**Figure 2:**
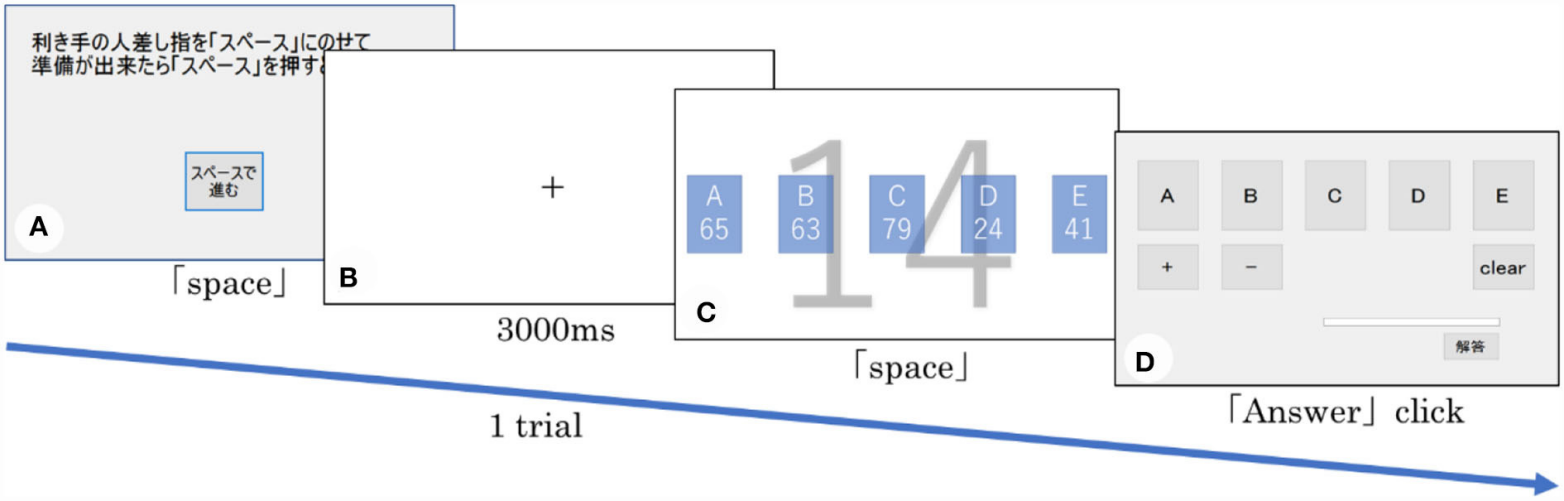
The task flow. **(A)**, **(C)**, and **(D)** are presented in the preparation, thought, and reporting phases, respectively. In **(B)**, the fixation point was presented in the center of the screen.

We used a modification of the water jar task (Luchins, 1942) to examine whether differences in the distribution of attention between the finders and the non-finders could be observed in trials using the trained procedure (Figures 1, 2). No special knowledge was required to solve this task. Consequently, it was possible to examine the differences between the finders and the non-finders independent of their expertise.

In Figure 1, the squares A–E represent five water jars, and the capacity of each is given a number (A = 65, B = 63, C = 79, D = 24, E = 41). The participants were requested to calculate the water quantity (14) indicated on the screen's background using these five water jars. In Figure 1, the solution is obtained by using C – D – E (79 – 24 – 41) and C – A (79 – 65). The former solution corresponds to the trained procedure and the latter to an alternative one.

The task consisted of induction (1st to 3rd) set (4th to 8th), critical (9th to 58th), and test trials (59th and 60th). The induction and set trials were those that could be solved using only the trained procedure. The induction trials were designed to encourage participants to discover a trained procedure. In these trials, the numbers D and E divisible by 10 were used to guide the participants to the trained procedure with certainty (e.g., C = 71, D = 40, and E = 20; the target number presented on the background = 11). The set trials were designed to repeatedly learn that the trained procedures could be used to solve problems. These trials were adjusted for the difficulty in applying the suboptimal procedure for C – D – E to be equal to that in the critical trials (all 2 digits, no carry and borrow, and indivisible by 10).

The critical trials were those that could be solved using the trained and alternative procedures. Participants who responded with C – D – E (trained procedure) at least once in the critical trial and found C – A (alternative procedure) were defined as the finder. In order to discuss the differences in the problem-solving process between the finders and non-finders, it was necessary to design the tasks in such a way that at least some participants without specialized knowledge could find alternative procedures. In Luchins (1942) water jar task, two critical trials were included, and in such a setting, most participants were unable to find an alternative procedure. Therefore, we conducted a preliminary experiment to investigate how many participants could find an alternative procedure by increasing the number of critical trials to 50. As a result, by increasing the number of trials to 50, 55% (18 participants) of the total participants (33 participants) found the alternative procedure. Therefore, in this study, we set the number of critical trials to 50.

In addition, the last two trials (59th and 60th) could be solved only with the alternative procedure. These trials were used as inspection trials to determine whether the participants engaged in the task earnestly. In the 59th trial, the result of C – D – E was set to 1 less than the background target number. In the 60th trial, D was set to a number greater than C. Therefore, clearly, the problem could not be solved using C – D – E as the trained procedure in the critical trials. If the participants engaged in the task earnestly, they would find that the formula C – D – E did not work to obtain the target amount of water. Thereby, the participants who answered C-D-E in the 60th trial of the task were excluded from the analysis.

### Apparatus

A Tobii T60 Eye Tracker (17-inch monitor, sampling rate 60 Hz) manufactured by Tobii Technology Co. was used for the task presentation and measurement of eye movements. Although we used our own program for stimulus presentation, all data measurement was controlled by Tobii T60 Eye Tracker, which was sufficiently accurate for measuring eye movements during the task. The screen resolution was 1280 × 1024 px and the screen size was 34.0 × 27.2 cm. The participants' heads were fixed 60 cm from the display by placement on a chin stand.

### Procedure

First, the participants confirmed the experimental procedures in practice. Before the main task, we calibrated the eye movement device. The participants conducted all of the trials at their own pace. Figure 2 shows a series of screenshots from each trial. In each trial, the task progressed in the following order: (1) preparation, (2) thought, and (3) reporting. During the preparation phase, the screen presented in Figure 2A was displayed and, when the participants were ready, they were required to press the space key using their index finger that displayed a crosshair at the center of the screen (Figure 2B). After 3,000 ms, the thought phase initiated automatically; one target and five jar numbers were displayed, as shown in Figure 2C. The participants were requested to press the space bar immediately after finding a solution. The time from the beginning of this thought phase to the pressing of the space bar was measured as the response time. Eye movements were assessed during this phase. In our study, the participants who discovered the alternative procedure after the 10th trial was called the finder group, because they found this solution after solving the critical trial with the trained procedure at least once. Participants who did not find the alternative procedure by the end of the critical trial were called the non-finder group. During the reporting phase, the participants reported a calculation formula by operating a mouse; moreover, they could not review the task screen presented during the thought phase (Figure 2D). After all the trials were completed, we conducted interviews to confirm whether they had reported the alternative procedure during the trial at which they had initially found it.

### Analysis and prediction

In the analysis, eye movement measurements were used to measure the distribution of attention. Specifically, two eye movement indices were used. The first index was the center of gravity of the gaze point in each trial. Specifically, the average of the x coordinate (horizontal axis) of all the gazes sampled in each trial was employed. The center of the screen corresponded to zero, and the positive and the negative value corresponded to the right (max 640 px) and the left direction (min – 640 px), respectively. The center on the horizontal axis was located at the water jar C's midpoint. By referring to this index, it was possible to assess whether the participants' distribution of attention was biased to the left or the right side. In the task, information for the trained and alternative procedure was presented on the right and the left side, respectively, allowing us to examine how much of the distribution of attention was biased toward either of the two solutions.

**Figure 3:**
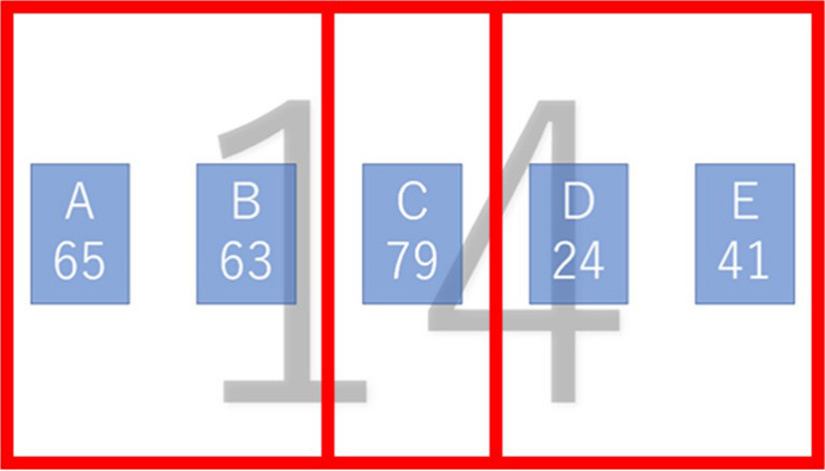
Image of the analysis areas (From the left, the L, C, and R areas).

For the second index, the ratio of fixation on each area in each trial (fixation duration rate: FDR) was used. To calculate this index, the screen presented in the thought phase was divided into three areas: right (R), center (C), and left (L) (Figure 3). Thus, we interpreted the R and L areas as part of the trained and alternative procedures, respectively. In each trial, we calculated the percentage of time during which fixation was observed in each area. The fixation time reflected the amount of information processing in that area (e.g., Frazier and Rayner, 1982). From this point of view, the FDR was an indicator of the ratio of attention focused on the information in each area within the trial. Hence, it was calculated for the areas of the trained and alternative procedure. The FDR also reflects the distribution of participants' fixations, that is, the degree to which they fixated on each region when the screen was divided into three regions.

To examine the distribution of attention during the phases using the trained procedure, usage of the trained procedure had to be ensured. Therefore, we examined the differences in two eye movement indices between finders and non-finders in the trials prior to the finding reporting trial, in which we could confirm from the participants' responses that the trained procedure was used. We defined the first trial in which finders found the alternative procedure as t and defined the one preceding t trial as a t-1 trial, and the one preceding t-1 trial as a t-2 trial. By this definition, t-1 and t-2 trials were the trials prior to the trial finding the alternative procedure. Thus, if there was a difference in the eye movement index in these trials, it could be argued that there was a difference in the distribution of attention between the finder and the non-finder from the point when the problem was being solved with the trained procedure.

The non-finder group did not have a finding reporting trial. Therefore, in order to compare the eye movement indices of the finders and non-finders, the number of trials of the non-finders used for comparison had to be matched to the finders. It was expected that the more trials that successfully solved the problem using a trained procedure, the stronger the fixation on the trained procedure would be (Gardner and Runquist, 1958; Crooks and McNeil, 2009). Therefore, we calculated an index of eye movements for the non-finders so that the number of trials to be compared would be the same for both groups. Specifically, we calculated the eye movement indices of the non-finder group corresponding to those of the finder group using the following procedure: First, we computed the average of the finding reporting trials of the latter's participants (referred to as t). Next, we made a linear approximation of the relationship between the number of trials in the critical trial and each eye movement index for each participant in the non-finder group. Finally, we estimated each eye movement index for each non-finder by substituting the average number of the finding reporting trials t into the approximation straight line. The index of eye movements in the pre- and post-finding reporting trials was also estimated by substituting the average finding reporting trials t ± 1, 2 into the approximation straight line.

In addition, in order to clarify whether there was a difference in the tendency of the inherent distribution of attention of the participants, we examined whether there was a difference in the eye movement indices in the first set trial. This allowed us to discuss whether the differences in the distribution of attention in the trials just before the finding reporting trial (t-1, t-2) were present from the beginning of the task. If differences in the distribution of attention existed not only just before the finding reporting trial, but also from the early phase of the task, the differences would be observed on the first set trial.

We made the following predictions related to differences in the distribution of attention between the finders and the non-finders. If the difference in the distribution of attention between the finders and the non-finders was present from the phase of using the trained procedure, differences in eye movement indices would be observed in the trials prior to the finding reporting trial t (t-1, t-2).

## Results

**Figure 4:**
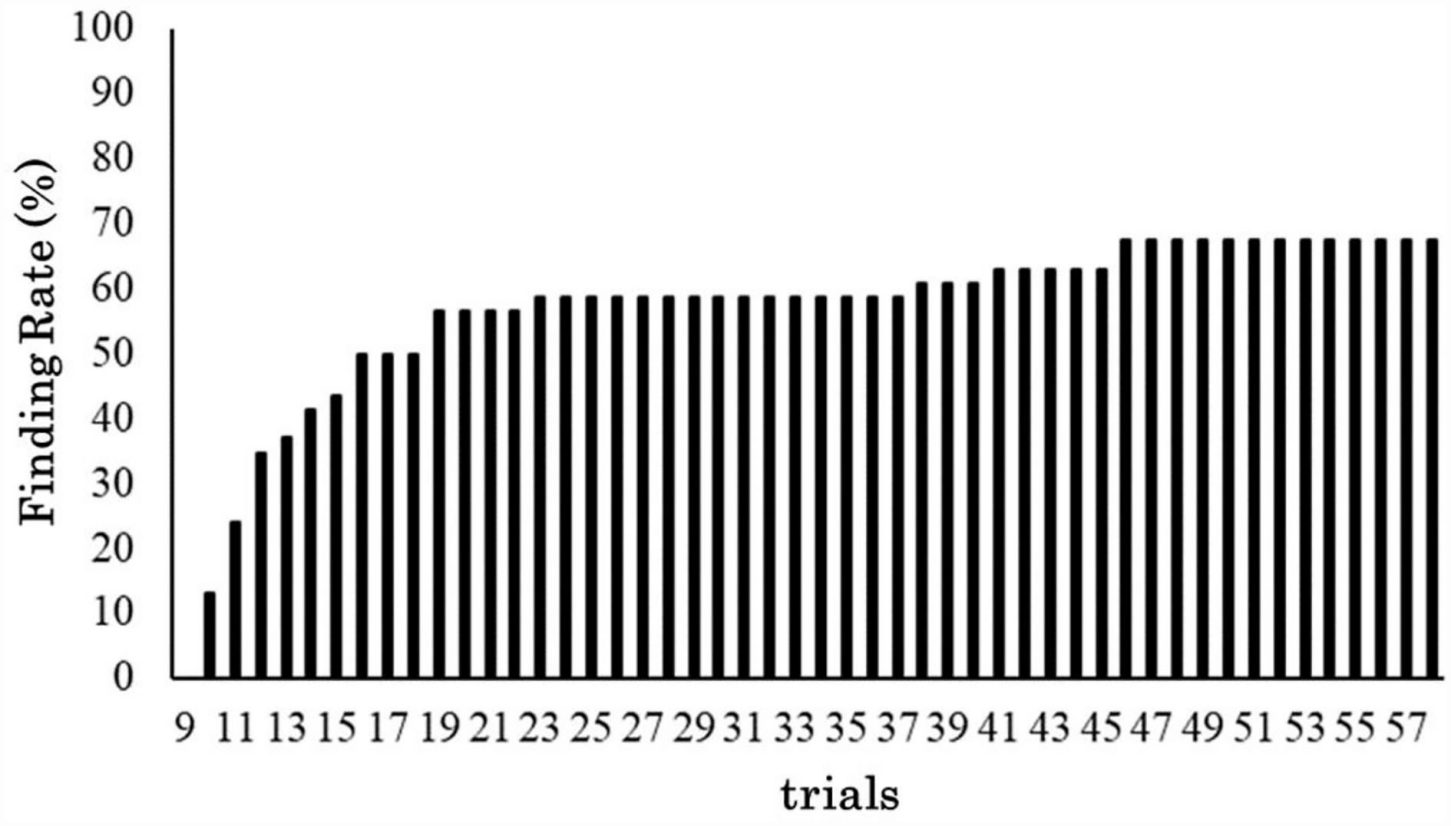
Transition of the rate of finding an optimal solution.

Among the 60 participants, one who discontinued the task and four who reported not answering C – A immediately in the trials where they actually found C – A (optimal solution) in the interview were excluded from the analysis. We considered that participants had learned the trained procedure when they answered with the trained procedure for at least one trial in the critical trial. Therefore, nine participants who found the alternative procedure during the 9th trial (the 1st one in the critical trials) were excluded from the analysis because they did not report the trained procedure in the critical trials. Consequently, 46 participants were included in the analysis. Figure 4 shows the transition of the rate of the participants who found an alternative procedure, indicating that 67% of the participants (31) found the alternative procedure before the last critical trial was completed.

### Differences in attention distribution prior to finding the alternative procedure

To examine the difference in the distribution of attention between the finders and non-finders, we compared the differences in the eye movement indices between the five trials around the finding reporting trial t. Therefore, we excluded five participants who reported the alternative procedure before the 11th trial; this was because the eye movement data of the two trials before the finding reporting trial during the critical trials were absent. Furthermore, we excluded four participants who had 50% or less valid eye movement data during the trials to be analyzed and one participant who answered incorrectly other than C – D – E during the five trials around the finding reporting trial t. Consequently, 21 participants who found the alternative procedure were classified as the finder group, while 15 who did not find it until the last trial was classified as the non-finder group. The average number of trials in which finders found the alternative procedure was 17.85.

#### Analysis using center of gravity of gaze point

**Figure 5:**
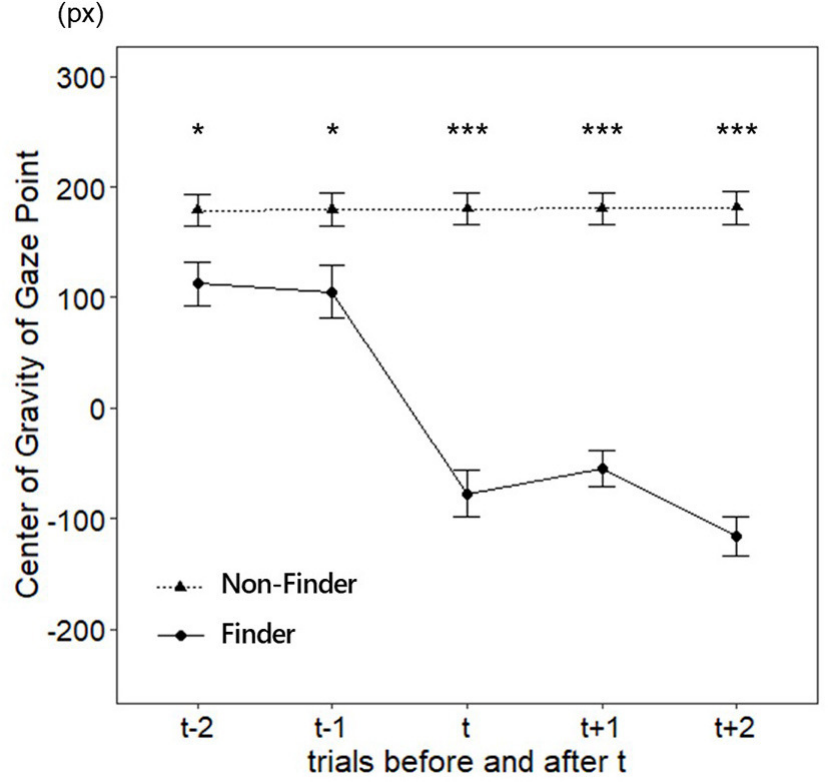
The center of gravity of the gaze point during the five trials around the finding reporting trial *t* (Right end = 640, Center = 0, Left end = −640). Error bars are *SE*. ***p < 0.001, **p < 0.01, *p < 0.05.

The center of gravity of the gaze point was used for the analysis. The mean and the standard error for each group are shown in Tables 1, 2, respectively. Initially, we assessed the difference in the center of gravity of the gaze point between the finder and non-finder before and after t (Figure 5). We performed a two-way mixed ANOVA with the groups as a between-factor (finder and non-finder) and trial as a within-factor (from t-2 to t + 2) to examine the differences in the center of gravity of the gaze point during the two trials around the finding reporting trial t. The results demonstrated that both the main effects of the group (*F*_(1, 34)_ = 83.07, *p* < 0.001, $\eta_{G}^{2}$ = 0.59) and the trial (*F*_(4, 136)_ = 30.26, *p* < 0.001, $\eta_{G}^{2}$ = 0.27) were significant. Additionally, we found a significant interaction between the two factors (*F*_(4, 136)_ = 31.29, *p* < 0.001, $\eta_{G}^{2}$ = 0.28).

**Table 1:** The mean (*SE*) of the eye movements for each group in the first set trial.

	**Finder mean**	**Non-finder mean**
	**(*SE*)**	**(*SE*)**
Center of Gtavity of GazePoint (px)	97.85 (14.31)	99.09 (17.18)
L area's FDR (%)	14.03 (2.11)	16.26 (3.13)
C area's FDR (%)	43.69 (2.13)	41.33 (1.85)
R area's FDR (%)	42.28 (1.84)	42.41 (2.63)

**Table 2:** Basic statistics of the simple main effect during the five trials around the finding reporting trial *t*.

		**Finder mean (*SE*)**	**Non-finder mean (*SE*)**	***df***	***F-*ratio**	***p***	** $\eta_{G}^{2}$ **
Center of Gravity of GazePoint (px)	t – 2	112.28 (19.89)	178.76 (14.67)	1, 34	6.22	0.018	0.15
	t – 1	105.29 (19.89)	179.38 (14.65)	1, 34	5.64	0.023	0.14
	t	−77.38 (20.77)	180.01 (14.63)	1, 34	87.16	<0.001	0.72
	t + 1	−54.67 (16.50)	180.63 (14.62)	1, 34	103.45	<0.001	0.75
	t + 2	−116.18 (17.84)	181.26 (14.62)	1, 34	147.44	<0.001	0.81
L area's FDR (%)	t – 2	11.07 (2.95)	3.05 (0.50)	1, 34	5.18	0.029	0.13
	t – 1	12.27 (3.37)	2.97 (0.49)	1, 34	5.33	0.027	0.14
	t	32.48 (2.32)	2.89 (0.48)	1, 34	113.25	<0.001	0.77
	t + 1	31.17 (1.93)	2.81 (0.46)	1, 34	148.92	<0.001	0.81
	t + 2	34.86 (2.64)	2.73 (0.45)	1, 34	103.57	<0.001	0.75
C area's FDR (%)	t – 2	48.14 (2.68)	46.58 (2.85)	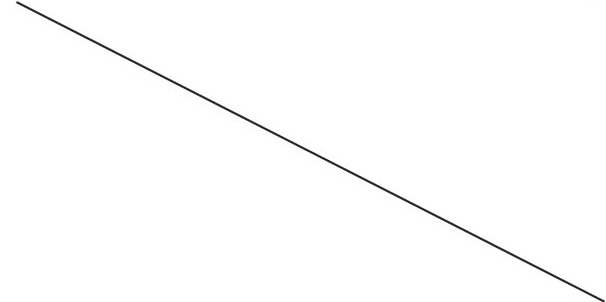
	t – 1	45.51 (2.65)	46.61 (2.83)		
	t	46.64 (2.19)	46.63 (2.82)		
	t + 1	43.67 (3.26)	46.65 (2.81)		
	t + 2	52.13 (3.86)	46.67 (2.80)		
R area's FDR (%)	t – 2	40.78 (2.52)	50.37 (2.75)	1, 34	6.42	0.016	0.16
	t – 1	42.22 (2.60)	50.43 (2.74)	1, 34	4.53	0.040	0.12
	t	20.87 (2.60)	50.48 (2.73)	1, 34	59.17	<0.001	0.64
	t + 1	25.16 (2.92)	50.54 (2.72)	1, 34	37.40	<0.001	0.52
	t + 2	13.00 (2.79)	50.60 (2.71)	1, 34	87.44	<0.001	0.72

The purpose of this study was to confirm whether there was a difference in the distribution of attention during the trials before the finding reporting trial t between the finders and non-finders. Therefore, we examined the simple main effects between the two groups in each trial before and after the finding reporting trial t. The results indicated that significant differences were found, not only in the t trial and after the t trial, but also in those before it (the statistics are summarized in Table 2).

Subsequently, we examined whether there were differences in the distribution of attention between the finders and non-finders from the early phase of the task. Therefore, we compared the center of gravity of gaze points in the first set trial between groups using Welch's *t*-test. Results showed no significant difference in the center of gravity of gaze point between the finders and non-finders (*t*_(30.05)_ = 0.06, *p* = 0.956, *r* = 0.01).

However, the t-test did not confirm that there was no difference. The two-side test (TOST) was conducted to examine the equivalence between the finders and non-finders. As a prerequisite for the TOST, bounds were set (“equivalence bounds”) on how much difference should be considered equivalent. We employed the smallest effect size of interest (SESOI) for the equivalence bounds, referring to Lakens (2017). In the absence of a clear theoretical background to define it, the SESOI has the smallest effect size (Cohen's *d* in the case of the *t*-test) when the sample size, the power (1 – β), and the α error probability were fixed. We calculated the minimum Cohen's *d* that rejected the null hypothesis when the sample size of each group was (21 finders and 15 non-finders), 1 – β = 0.80, and α = 0.05 (Cohen's *d* = 0.97). Subsequently, we performed the TOST using Cohen's *d* as the equivalence bounds. The results showed a significant equivalence between the two groups (*t*_(34)_ = 2.81, *p* = 0.004, upper: *t*_(34)_ = 2.81, *p* = 0.003, lower: *t*_(34)_ = −2.92, *p* = 0.004).

#### Analysis using FDR

We analyzed the FDR to examine the information search of the participants from a different perspective. Its mean and standard errors for all areas are shown in Tables 1, 2.

**Figure 6:**
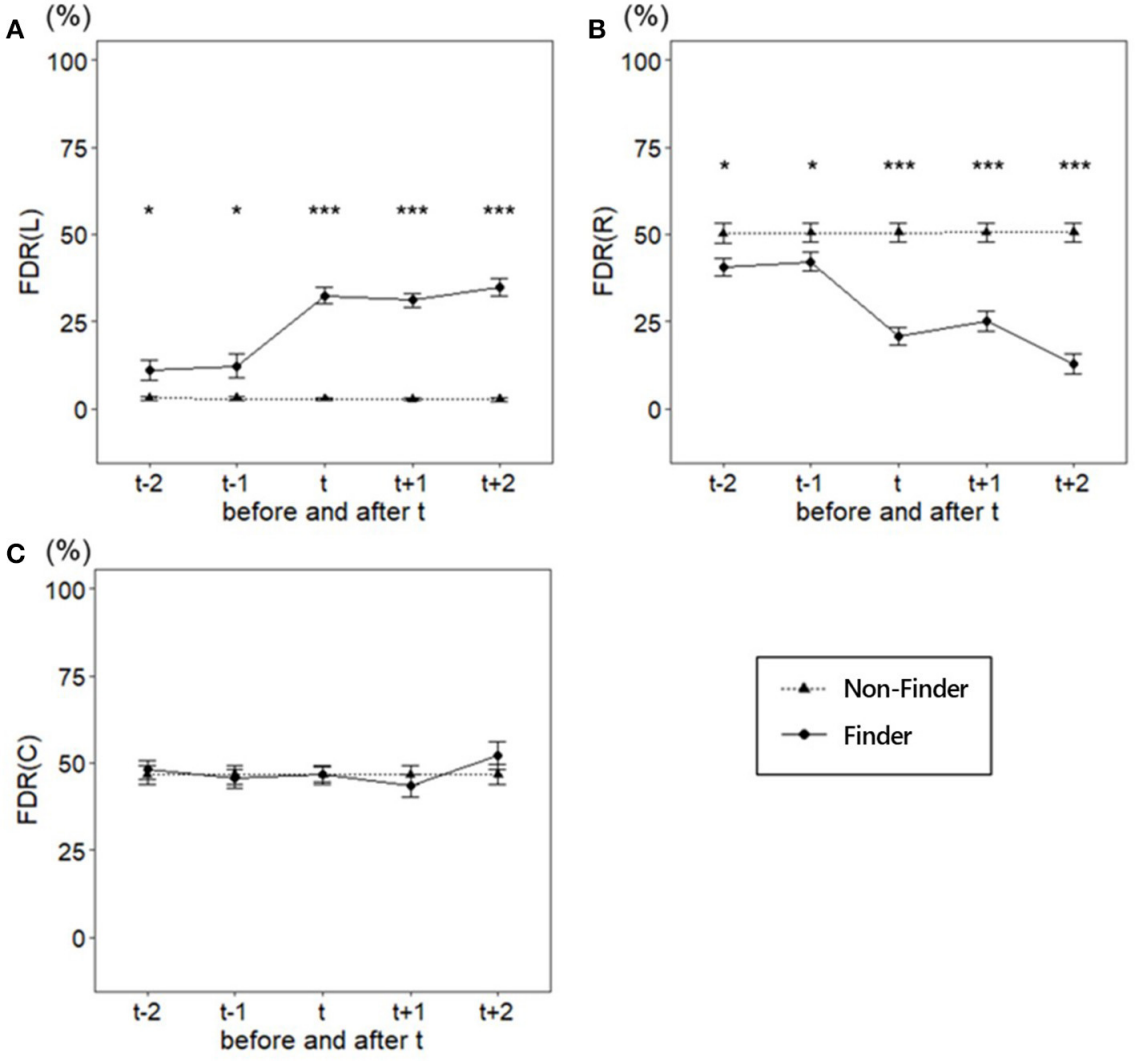
Fixation Duration Rate during the two trials before and after the finding reporting trial *t*. **(A)** FDR in L area, **(B)** FDR of R area, **(C)** FDR of C area. Error bars are *SE*. ***p < 0.001, **p < 0.01, *p < 0.05.

We repeated the two-way mixed ANOVA with groups as a between-factor (finder and non-finder) and trial as a within-factor (from *t* – 2 to *t* + 2) to examine the differences in the FDR of the L area during the five trials around the t trial (Figure 6A). The results showed that both the main effects of group (*F*_(1, 34)_ = 85.84, *p* < 0.001, $\eta_{G}^{2}$ = 0.57) and trial (*F*_(4, 136)_ = 21.49, *p* < 0.001, $\eta_{G}^{2}$ = 0.24) were significant. Furthermore, we found a significant interaction (*F*_(4, 136)_ = 22.33, *p* < 0.001, $\eta_{G}^{2}$ = 0.24). Similar to the analysis of the center of gravity of the gaze point, we assessed the simple main effects between the groups during each of the trials before and after the finding reporting trial t; the results are displayed in Table 2. The results showed that the FDR in the L region was significantly greater in the finders than in the non-finders, not only in the t and after t trials but also before the t trials.

We then analyzed the FDR of the R area, which was the area of the optimal solution. To examine the difference in the FDR of the R area during the five trials around the t trial (Figure 6B), we performed a two-way mixed ANOVA with a group as a between-factor (finders and non-finder) and trial as a within-factor (from t – 2 to t + 2). Consequently, both the main effects of group (*F*_(1, 34)_ = 60.46, *p* < 0.001, $\eta_{G}^{2}$ = 0.48) and trial (*F*_(4, 136)_ = 17.27, *p* < 0.001, $\eta_{G}^{2}$ = 0.20) were significant. Additionally, we found a significant interaction (*F*_(4, 136)_ = 17.72, *p* < 0.001, $\eta_{G}^{2}$ = 0.20). We assessed the simple main effects between the groups in each of the trials around the finding reporting trial, as presented in Table 2. The results showed that the FDR in the R region was significantly smaller in the finder than in the non-finder, not only in the t and after t trials but also before the t trials.

Finally, we analyzed the FDR of the C area, which was the area related to both solutions. If the FDR reflected the amount of information processing correctly, then there would be no significant difference in FDR between the groups during the five trials around the finding reporting trial t in the C area because the C area is associated with both solutions. To examine differences in the FDR of the C area during the five trials around the t trial, we performed a two-way mixed ANOVA with a group as a between-factor (the finders and the non-finders) and a trial as a within-factor (from t – 2 to t + 2; Figure 6C). There were no significant effects (main effect of group (*F*_(1, 34)_ = 0.04, *p* = 0.851, $\eta_{G}^{2}$ = 0.00) main effect of trial (*F*_(4, 136)_ = 0.98, *p* = 0.421, $\eta_{G}^{2}$ = 0.01), and interaction (*F*_(4, 136)_ = 0.97, *p* = 0.428, $\eta_{G}^{2}$ = 0.01).

Subsequently, we examined whether there were differences in each FDR between the finders and non-finders from the early phase of the task. Therefore, we compared each FDR in the first set trial between groups using Welch's *t*-test. Results showed no significant difference in each FDR between the finders and non-finders (L area: *t*_(25.82)_ = 0.59, *p* = 0.560, *r* = 0.12; R area: *t*_(26.63)_ = 0.04, *p* = 0.968, *r* = 0.01; C area: *t*_(33.94)_ = 0.84, *p* = 0.409, *r* = 0.14).

To assess the equivalence of each FDR in the first set trial between the finders and non-finders, we performed the TOST. We used the SESOI (Cohen's *d* = 0.97) for equivalence bounds based on the same criteria as that of the center of gravity of the gaze point analysis (Lakens, 2017). A significant equivalence was found between the two groups in the L (*t*_(34)_ = 2.26, *p* = 0.015, upper: *t*_(34)_ = −3.48, *p* < 0.001, lower: *t*_(34)_ = 2.26, *p* = 0.015), C (*t*_(34)_ = −2.07, *p* = 0.023, upper: *t*_(34)_ = −2.07, *p* = 0.023, lower: *t*_(34)_ = 3.66, *p* < 0.001), and R areas (*t*_(34)_ = 2.83, *p* = 0.004, upper: *t*_(34)_ = −2.91, *p* = 0.004, lower: *t*_(34)_ = 2.83, *p* = 0.003).

#### *Post hoc* analysis

The results of the previous analyses indicated that there were differences in the trials immediately before finding (t-1, t-2), which were not seen at the beginning of the task (the first set trial). Therefore, we conducted a *post hoc* analysis of how the differences in the distribution of attention between participants occurred, which was not the initial question but was not seen at the beginning of the task. Specifically, we discuss how the distribution of attention changed for the trial immediately before the finding compared to the beginning of the task.

Therefore, to examine the difference in changes in the center of gravity of the gaze point from the first set trial to t-2 and t-1 trials between finders and non-finders, we performed a two-way mixed ANOVA with a group as a between-factor (finders and non-finders) and trial as a within-factor (the first set trial, t-2 and t-1). The results demonstrated that both the main effects of the group (*F*_(1, 34)_ = 4.78, *p* = 0.036, η*G*^2^ = 0.08) and the trial (*F*_(2, 68)_ = 7.09, *p* = 0.002, η*G*^2^ = 0.07) were significant. Additionally, we found a significant interaction between the two factors (*F*_(2, 68)_ = 4.11, *p* = 0.021, η*G*^2^ = 0.04). We then tested for a simple main effect of trial for each group, the bias in distribution of attention was significantly larger for the non-finder group as the trial progressed (*F*_(2, 28)_ = 14.03, *p* < 0.001, η*G*^2^ = 0.30), whereas there was no such significant trend for the finder group (*F*_(2, 40)_ = 0.26, *p* = 0.773, η*G*^2^ = 0.00).

## Discussion

### Timing of observed difference in the distribution of attention between finders and non-finders

The purpose of this study was to determine whether a difference in the distribution of attention between the finders and non-finders in the t-2 and t-1 trials existed during which the finders were solving the problem in a trained procedure. The results of the experiment showed that there was a difference in the bias of the distribution of attention between the two groups. This indicates that the finders and the non-finders differ, not only in whether or not they could focus their attention on other procedures just before discovering an alternative procedure but also in the degree of fixation and the information to which attention is directed during the phase of solving a problem using the trained procedure. The importance of the present results is the evidence that the difference in the distribution of attention between finders and non-finders is present even before the discovery of the alternative procedure. This is evident even when a clear distinction is made between pre- and post-finding.

Specifically, analysis using the center of gaze point showed that, although the finders also biased their attention on the area regarding the trained procedure, the degree of bias was smaller than that of the non-finders. Analysis of the R and L areas revealed that the finders paid more attention to information unrelated to the trained procedure, such as water jars A and B, and less attention to information related to the trained procedure, such as water jars D and E, compared to the non-finders. As for the overall distribution, participants' fixations were mostly biased toward the center and L area in the non-finder, whereas the finder fixated less on the L area and more on the R area than the non-finder. These results indicate that the finder pays less attention to the trained procedure and more attention to an irrelevant area before the finding of the trained procedure, both for the center of gravity and for the distribution. In other words, the finders paid more attention to the information that was not necessary to respond using the trained procedure than the non-finders. Despite choosing a familiar procedure, a certain degree of attention to other information can work to one's disadvantage in the context of general problem solving (Gilhooly and Fioratou, 2009; Wiley and Jarosz, 2012) because it creates a distraction for effective solutions. Several previous studies have demonstrated that distraction caused by cognitive loads, such as pressure, promotes the discovery of alternative procedures (Beilock and DeCaro, 2007; Ricks et al., 2007; DeCaro, 2018). The present results provide new empirical evidence that attentional distraction promotes the discovery of alternative procedures under the Einstellung effect in the situation in which continuous use of the trained procedure works to a disadvantage.

### How did the difference in the distribution of attention between finders and the non-finders occur?

This study also aimed to examine whether differences could be observed in the participants' inherent tendency to distribute attention. The results of the experiment showed that there was no significant difference in the distribution of attention between the finders and the undiscovered in the first trial of the set trial, which was a trial to train the trained procedure. The important point here is that the equivalence hypothesis that there is no difference was supported. This result indicates that a difference in the participants' inherent tendency to the distribution of attention does not exist between the finders and the non-finders even before they begin the task.

The absence of differences in the participants' inherent distribution of attention means that the differences just before the finding reporting trial arose through repeated experience of trials in which problem solving was successful using the trained procedure. Previous research demonstrated that the more repeated the training trials that the trained procedure can solve the problem, the stronger the fixation on the trained procedure becomes (Crooks and McNeil, 2009). As both the finders and non-finders were solving problems with the trained procedure until the t trial, it can be considered that they were repeating the training trial.

How the difference emerged through repeated training can be discussed by comparing the eye movement indices between the first set trial and t-2 and t-1 trials. The eye movement indices for the first set trial and the t-2 and t-1 trials showed that the bias in the distribution of attention to the trained procedure was enhanced from the first set trial to t-2 and t-1 trials in both the finders and non-finders (Tables 1, 2). The degree of that enhancement was greater for the non-finders than for the finders. Indeed, *post hoc* analyses showed that for the non-finder, the distribution of attention was significantly biased in the direction of the placement of the trained procedure in the trial immediately before discovery (t-1, t-2) than at the beginning of the task (first set trial), but no such difference was observed for the finder.

These results mean that the non-finders' bias in the distribution of attention became stronger through repeated training trials, whereas the finders' bias was not so strengthened. This suggests that the finders had more resistance to the enhancement of fixation caused by the success of problem solving using a familiar procedure than non-finders. In other words, the difference in the distribution of attention between the finders and the non-finders might be caused by the difference in resistance to the enhancement of fixation to the trained procedure by repeated training trials. However, the validity of this interpretation requires further detailed examination in the future, since these results were provided by additional *post hoc* analyses to interpret the experimental results. Therefore, future work is required to directly examine the change in the distribution of attention in the process of enhanced mental set and the process of acquiring a trained procedure.

### Finding an alternative procedure in situations when the problem “cannot” be solved using the trained procedure

In this study, we confirmed that a difference in the distribution of attention was observed between the finder and the non-finder in a situation in which the problem can be solved using a trained procedure. The discovery of an alternative procedure in situations in which the problem cannot be solved using a familiar procedure has been explained based on the feedback of failure (Chesney et al., 2013; Sheridan and Reingold, 2013). Specifically, the failure of the familiar procedure inhibits the exploration related to that procedure and leads to the finding of an alternative, better solution. The present results indicate that even if problem solving using the trained procedure does not fail, about half of the participants diverted their attention from the trained procedure and found the alternative procedure. This study did not examine what factors caused this difference in the distribution of attention. However, based on the setting of our task, it is suggested that factors other than failure may have caused this difference. In future studies, it is necessary to examine factors other than failure that motivate people to reconsider their procedures and spontaneously explore other methods in situations where the problem “can” be solved by a trained procedure.

In examining factors other than failure that prompt rethinking of automated procedures such as the trained procedure, findings about meta-reasoning may provide useful insights. In the domain of decision making and reasoning, it is known that one's judgments and decisions are implicitly evaluated and that these evaluations influence the control of one's judgments (Ackerman and Thompson, 2017). For example, it has been demonstrated that the more fluent one's judgment or decision-making, the more likely one is to feel one's intuitive judgments as being more rightness, and consequently, the less likely one is to change one's judgment (Alter et al., 2007; Thompson et al., 2013). These evaluations may provide a cue to divert attention away from the trained procedure under the Einstellung effect in situations where there is no feedback of failure. For example, those who believe the trained procedure is correct may be more likely to focus their attention on information related to it, while those who do not believe the trained procedure is correct may be more likely to divert their attention from it.

In addition, research on covariation-learning has shown that other procedures can be learned even in situations where the participants know the procedure that can solve the problem (Schuck et al., 2015, 2022; Gaschler et al., 2019). These studies deal with situations in which participants are required to make a response corresponding to a stimulus with covariant two features. Furthermore, here, in a situation where the correspondence between one feature and the response is acquired through instruction, how the relationship between the other feature and the response learned is examined (Schuck et al., 2015; Gaschler et al., 2019). For example, Schuck et al. (2015) indicated that learning about such covariant features progressed even in situations where no conflict arises in the known correspondence. This implies that learning for alternative procedures progresses even when participants respond correctly based on known procedures. These studies deal with different situations from the Einstellung task in that known procedures are given by instructions and the focus is on the performance of learning about alternative procedures, that is, whether covariant features can be used when the indicated correspondence does not work. Meanwhile, they seem to share a common problem with the present study because they deal with the question of whether a shift to an alternative procedure occurs in a situation that can solve a problem with a previously acquired procedure. From this point of view, it is important to examine whether the finding of an alternative procedure in the Einstellung task is a result of some kind of learning, and if so, how such learning proceeds.

In future studies, we will examine how people evaluate trained procedures and learn alternatives and the influence of such evaluations and learning on the exploration and discovery of an alternative procedure.

## Conclusion

This study examined whether the difference in the distribution of attention between the finders and the non-finders of the alternative procedure is observed from the phase of solving the problem using the trained procedure. First, we divided the discovery of the alternative procedure by whether the trained procedure failed or succeeded. Next, we approached the characteristics of the finder's distribution of attention in situations where problem solving using a trained procedure was successful, which has been little examined in previous research. Our results demonstrated with empirical evidence that, compared to the non-finders, the finders paid more attention to information unrelated to the familiar procedure acquired through knowledge and experience, even when using a familiar procedure. We also confirmed that this difference does not exist from the beginning of the task, but emerges during repeated use of familiar procedures. These findings indicate that to find an alternative procedure, it is important to not only divert attention from a familiar procedure just before the discovery but also pay a certain amount of attention to information unrelated to the familiar procedure even when the familiar procedure is functioning well.

## Data availability statement

The raw data supporting the conclusions of this article will be made available by the authors, without undue reservation.

## Ethics statement

The studies involving human participants were reviewed and approved by Cognitive Science Course, Department of Informatics, Nagiya University. The patients/participants provided their written informed consent to participate in this study.

## Author contributions

All authors listed have made a substantial, direct, and intellectual contribution to the work and approved it for publication.

## Funding

This work was supported by the JSPS KAKENHI [grant numbers 22H03912].

## Conflict of interest

The authors declare that the research was conducted in the absence of any commercial or financial relationships that could be construed as a potential conflict of interest.

## Publisher's note

All claims expressed in this article are solely those of the authors and do not necessarily represent those of their affiliated organizations, or those of the publisher, the editors and the reviewers. Any product that may be evaluated in this article, or claim that may be made by its manufacturer, is not guaranteed or endorsed by the publisher.
